# Microstructure and Biological Properties of Electrospun In Situ Polymerization of Polycaprolactone-Graft-Polyacrylic Acid Nanofibers and Its Composite Nanofiber Dressings

**DOI:** 10.3390/polym13234246

**Published:** 2021-12-03

**Authors:** Yi-Jen Huang, Chien-Lin Huang, Ruo-Yu Lai, Cheng-Han Zhuang, Wei-Hao Chiu, Kun-Mu Lee

**Affiliations:** 1 Department of Fiber and Composite Materials, Feng Chia University, Taichung 40724, Taiwan; yijenhua@gmail.com (Y.-J.H.); judy91206@gmail.com (R.-Y.L.); skyforest1316@gmail.com (C.-H.Z.); 2 Center for Green Technology, Chang Gung University, Taoyuan 33302, Taiwan; weihao.chiu@gmail.com; 3 Department of Chemical and Materials Engineering, Chang Gung University, Taoyuan 33302, Taiwan; 4 Department of Pediatrics, Chang Gung Memorial Hospital, Linkou, Taoyuan 33305, Taiwan

**Keywords:** electrospinning, in situ polymerization, polycaprolactone, poly(acrylic acid), grafting, nanofiber, graphene oxides, photoinitiator, hydrophilic, wound healing, ethylene glycol dimethyl acrylate, poly[2-(tert-butylaminoethyl) methacrylate]

## Abstract

In this study, polycaprolactone (PCL)- and poly(acrylic acid) (PAA)-based electrospun nanofibers were prepared for the carriers of antimicrobials and designed composite nanofiber mats for chronic wound care. The PCL- and PAA-based electrospun nanofibers were prepared through in situ polymerization starting from PCL and acrylic acid (AA). Different amounts of AA were introduced to improve the hydrophilicity of the PCL electrospun nanofibers. A compatibilizer and a photoinitiator were then added to the electrospinning solution to form a grafted structure composed of PCL and PAA (PCL-*g*-PAA). The grafted PAA was mainly located on the surface of a PCL nanofiber. The optimization of the composition of PCL, AA, compatibilizer, and photoinitiator was studied, and the PCL-*g*-PAA electrospun nanofibers were characterized through scanning electron microscopy and ^1^H-NMR spectroscopy. Results showed that the addition of AA to PCL improved the hydrophilicity of the electrospun PCL nanofibers, and a PCL/AA ratio of 80/20 presented the best composition and had smooth nanofiber morphology. Moreover, poly[2 -(tert-butylaminoethyl) methacrylate]-grafted graphene oxide nanosheets (GO-*g*-PTA) functioned as an antimicrobial agent and was used as filler for PCL-*g*-PAA nanofibers in the preparation of composite nanofiber mats, which exerted synergistic effects promoted by the antibacterial properties of GO-*g*-PTA and the hydrophilicity of PCL-*g*-PAA electrospun nanofibers. Thus, the composite nanofiber mats had antibacterial properties and absorbed body fluids in the wound healing process, thereby promoting cell proliferation. The biodegradation of the PCL-*g*-PAA electrospun nanofibers also demonstrated an encouraging result of three-fold weight reduction compared to the neat PCL nanofiber. Our findings may serve as guidelines for the fabrication of electrospun nanofiber composites that can be used mats for chronic wound care.

## 1. Introduction

Chronic skin wounds impose considerable medical burden, especially to individuals suffering from burns [1] and chronic skin ulcers [2,3,4]. A good skin wound dressing needs to satisfy the following requirements [5]: good tissue compatibility, which prevents toxicity or inflammation [6,7]; good moisture retention, which maintains the moist environment of the wound and promotes cell hydration [8]; and sufficient physical and mechanical durability, which ensures its integrity and prevents external bacterial infection due to material damage [9]. Polycaprolactone (PCL) is a polymer material widely used in biomedical applications, including wound healing, drug delivery [10], and bone regeneration [11]. PCL has good mechanical properties [12], biocompatibility [12], and slow biodegradability [13], showing potential as a material for chronic wound dressings [14]. However, PCL is a hydrophobic polymer and has limited practical applications. Therefore, many studies have modified the PCL surface to improve the hydrophilicity of PCL [15,16,17]. Some studies showed that increase in the hydrophilicity of PCL can promote the biodegradability of PCL [18].

One of the most popular polymers used in altering the hydrophilicity of PCL is PAA [19,20,21]. Owing to its good hydrophilicity, PAA has been widely used in biomaterials. It is an ionic polymer known for its pH-responsive behavior due to its polymer backbone with ionic pendant groups [22,23,24]. In addition, hydrogels made from PAA can absorb and retain large amounts of water in their structures without breaking down [22,25]. Therefore, PAA not only affords PCL high water uptake capacity but also couples the properties of hydrogels for developing wound dressings. Hydrogel-based wound dressings can aid wound healing while sustaining a moist surrounding and absorbing exudates [26]. Hydrogel-based dressings made from PAA are widely used owing to their pH-responsive behavior and water-swelling characteristics. However, the low mechanical strength of hydrogel-based dressings limits their application [27]. The grafting of PCL and PAA may be a means to couple the mechanical property of PCL and the hydrogel property of PAA. The grafting of PAA to polymers, such as poly(vinyl alcohol) (PVA) [28], poly(lactic acid) (PLA) [29], and poly(ethylene glycol) (PEG) [30] is a feasible method for modifying polymers. Guo et al. [31] synthesized core/shell-PS/PAA particles through photopolymerization and proposed a method for fabricating a PAA grafting polymer by using photoinitiators. The grafting of PAA and PCL was performed in some studies. Kim et al. [32] synthesized PAA-*g*-PCL copolymer and blended it with starch/PCL. Song et al. [33] synthesized PAA by grafting PCL through reversible addition-fragmentation chain transfer polymerization and ring-opening polymerization. Ata et al. [34] These studies provided the foundation for PAA and PCL grafting. Notably, PCL and PAA are immiscible, and a compatibilizer might be required for in situ polymerization, particularly in the blending of low-density polyethylene with polyamide-6 [35]. Although ethylene glycol dimethyl acrylate (EDGMA) is a cross-linking agent for preparing PCL and AA hydrogels, the function of EDGMA as a compatibilizer should be considered [36].

A vital determinant in chronic wound healing is the prevention of bacterial infections. Wound dressings with antibacterial features can eliminate bacteria on wound surfaces and diminish infection and inflammation. Hence, electrospun fiber mats permeated with antibacterial agents have become a major subject of interest in chronic wound healing [37,38]. Graphene nanosheets possess excellent antibacterial properties [39] and can accelerate cell differentiation [40] and are thus practical antibacterial materials for filling electrospun fiber mats [41]. Graphene nanosheets can be obtained through the hydrazine or thermal reduction of graphene oxide (GO) [42]. In this study, the GO was prepared with Hummers’ method. To improve the antibacterial ability of GO, cationic polymers, such as polyethyleneimine and poly[2-(tert-butylaminoethyl) methacrylate] (PTA), can be used to modify GO [43]. Huang et al. [44] showed that PTA grafted onto the surface of GO through free radical polymerization and atom transfer radical polymerization (ATRP) improve the antibacterial efficiency of GO. A composite nanofiber mat consisting of electrospun nanofibers and GO-*g*-PTA nanosheets prepared through ATRP showed good antibacterial ability and non-cytotoxicity.

**Figure 1:**
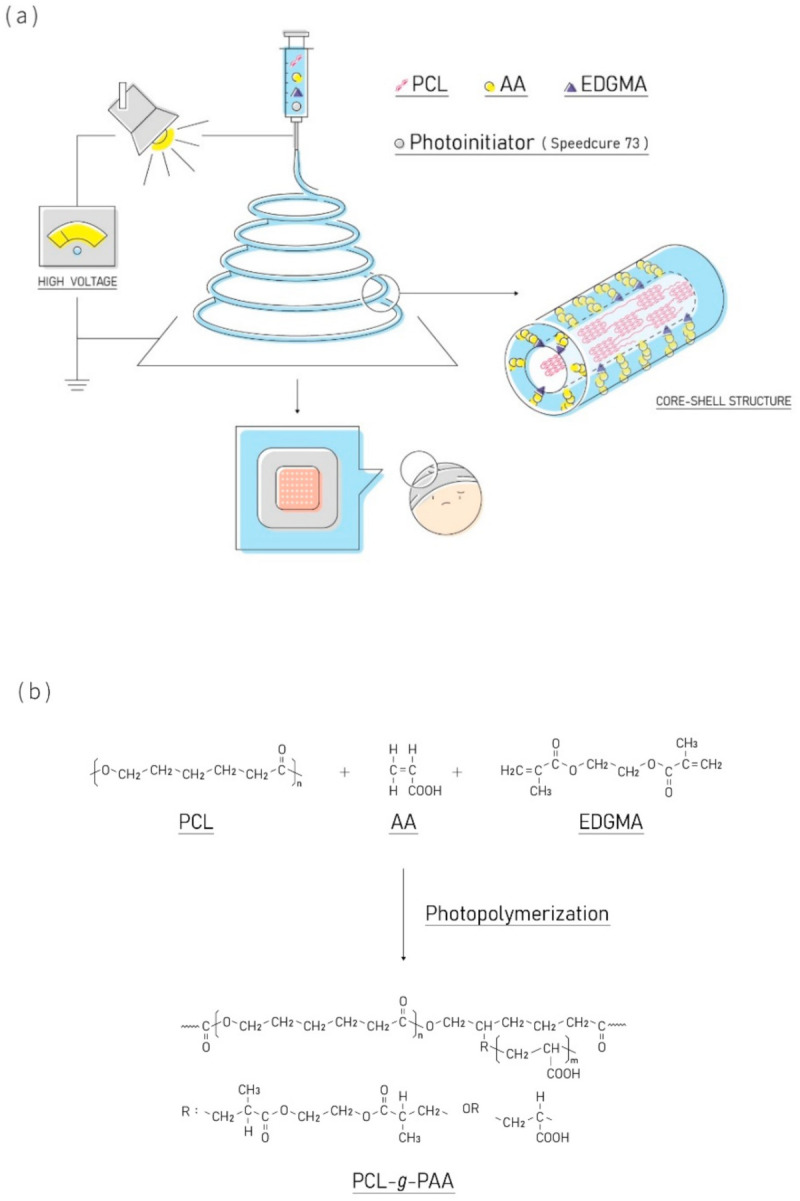
(**a**) the schematic diagram and (**b**) synthetic reaction scheme of the in situ grafting polymerization of PCL and PAA during electrospinning.

The grafting of PCL and PAA [32,33,34] and the electrospinning of PCL-*g*-PAA have been explored [45]. However, the in situ grafting polymerization of PCL and PAA during electrospinning has not been examined yet. This work investigated the optimization of in situ polymerization in terms of electrospinning solution properties and the relationship between compatibilizers and photoinitiators during grafting polymerization. Figure 1 shows the schematic diagram and synthetic reaction scheme of the in situ grafting polymerization of PCL and PAA during electrospinning. The antibacterial effects of the composite nanofiber mats fabricated through the coupling of GO-*g*-PTA nanosheets and PCL-*g*-PAA nanofibers were studied. The relationships among AA monomers, PCL polymers, compatibilizers, and photoinitiators during electrospinning were studied by examining molecular structural and morphological characteristics of the grafting nanofibers through ^1^H-NMR spectrum and scanning electron microscopy (SEM). The properties of electrospun nanofibers and composite nanofiber mats for chronic wound dressing applications were evaluated using contact angle measurements and biodegradation, cell viability, and antibacterial tests.

## 2. Materials and Methods

### 2.1. Materials

Polycaprolactone powders with a number average molecular weight of 80,000 g/mole, acrylic acid, and (2-tert-butylaminoethyl) methacrylate were supplied by Sigma-Aldrich, St. Louis, MO, USA. Dimethylformamide (DMF) and tetrahydrofuran were purchased from Echo Chemical Co. (Miaoli, Taiwan). Chloroform was supplied by J.T. Baker, Radnor, PA, USA. Copper(I) bromide (CuBr), 1,1,4,7,10,10-hexamethyl-triethylenetetramine, 2-bromopropionyl bromide, and EDGMA were supplied by Alfa Aesar, Ward Hill, MA, USA. Sulfuric acid (97% purity) was provided by Showa, Tokyo, Japen. Lastly, 2-hydroxy-2-methy-l-propiophenone (Speedcure 73; purity of 96%) was purchased from Avantor Inc., Radnor, PA, USA.

### 2.2. Preparation of the Electrospinning Solution

The electrospinning solution was prepared. The solvent was a mixture of chloroform and DMF with a volume fraction of 9–1, and the weight percentage of the solute was maintained at 7%. The solute was a mixture of AA and PCL in specified ratios. For the samples containing compatibilizer (EDGMA) and photoinitiator (Speedcure 73), a PCL/AA solution was mixed thoroughly, and then the compatibilizer and photoinitiator were added. To improve the antimicrobial ability of composite nanofiber mats, GO-g-PTA was used as an antimicrobial agent and was added to PCL-g-PAA nanofibers. GO and GO-*g*-PTA were synthesized with Hummers’ method. The specific process (ATRP) was mentioned in our previous study [44]. For samples containing GO-*g*-PTA, GO-*g*-PTA was mixed with chloroform and DMF in an ultrasonic mixer for 3 h, and then PCL and AA were added. The solution with GO-*g*-PTA, PCL, and AA was stirred for 12 h at room temperature, and a compatibilizer and a photoinitiator were added. Solution conductivity property (κ) was measured at 25 °C with an Oakion electrical conductivity meter (PC700, Vernon Hills, IL, USA). The viscosity of the solution was measured using a viscometer (DV-II+Pro, spindle 18, and cup 13R, AMETEK Brookfield, Middleboro, MA, USA) at 25 °C. The PTA percentage for GO-*g*-PTA was 22.3 wt%.

### 2.3. Electrospinning Process

Before electrospinning, electrospinning solutions were prepared in the absence of light. The prepared solutions were subjected to room-temperature electrospinning, and the needle size was *D_i_*/*D*_0_/length = 0.69 mm/1.07 mm/ 40 mm (where *D_i_* and *D*_0_ were the inner diameter and outer diameter of the needle, respectively). A black cloth was used to block the syringe and light source. The prepared solution was delivered to the needle by a syringe pump (Cole-Parmer) to the needle at controlled flow rate (*Q*). High electrical voltage (*V*) was applied to the spinneret by using a high voltage source (MECC, HVU-40P100, Fukuoka, Japan) to provide sufficient electric field for electrospinning. An aluminum plate (30.5 cm × 30.5 cm) was used as a collector for electrospun fibers, with a fixed distance of 14 cm from the tip to the collector for the construction of a needle-to-plate electrode configuration. Figure S1d the functioning domain for 7 wt% PCL electrospinning solution with various proportions of AA and GO-*g*-PTA. The functioning domains were the operating windows of electric field and flow rates that were required for a stable cone-jet mode. The lower- and upper-bound electric fields were denoted as *V_s_* and *V_us_*, respectively. Given the volatility of the solvent, a working distance (*H*) of 14 cm was used. At a specific *Q*, the operating windows (*V_us_*–*V_s_*) were the variations in the PCL solutions with various proportions of AA and GO-*g*-PTA. Based on the functioning domain for electrospinning PCL/AA and PCL/GO-*g*-PTA solutions (Figure S1, Supplementary Materials), a common but limited processing window for determining the effects of AA and GO-*g*-PTA proportions was available. Therefore, the determined *Q* and *V* were 0.4 mL/h and 9 kV, respectively, in the electrospinning PCL/AA, PCL/GO-*g*-PTA, and PCL/AA/GO-*g*-PTA solutions to demonstrate the effects of AA and GO-*g*-PTA on the fiber diameter and nanofiber morphology. Photo-induced polymerization was initiated by exposing the tip and the collector to visible light. After electrospinning, polymerization reaction was ensured by placing the nanofibers under a light source for 2 days. In the present study, the samples were designated according to their respective weight ratios, as presented in Table 1. The weight percentages for the photoinitiator and EDGMA were based on the weight percent of AA monomers. In Table 1, the symbol i in the sample code stands for the weight percentage of the photoinitiator, and the symbol c in the sample code stands for the weight percentage of the compatibilizer (EDGMA). The purpose of the addition of AA was to improve the hydrophilicity of PCL. Therefore, increase in the amount of AA was preferred. The nanofiber with a composition of PCL/AA 80/20 was the best option for further study because of its nanofiber morphology. The detailed nanofiber morphology is discussed in Section 3.1.

### 2.4. Determination of the Degree of Grafting

Unreacted components were removed through the purification of the nanofibers. The nanofibers were immersed in the DMF solvent in a weight ratio of 1/100 and stirred at 65 °C for 1 day. After stirring for 1 day, the mixture solution was precipitated dropwise into a 20-fold excess volume of methanol. Unreacted AA was purified through dissolution and precipitation, which were performed three times. The precipitated powders were dried in a vacuum oven at 40 °C for 72 h until the residual solvent was removed. The percentage of weight loss during purification was recorded. Acid–base titration was performed on the nanofibers for the study of grafting rate. Phenolphthalein (1 wt%) as an acid–base indicator was added to the nanofiber-containing DMF solution, and the mixture was stirred uniformly at 70 °C for 2 h and titrated with 0.03 N KOH solution. Then, the degree of grafting was calculated using Formulas (1) and (2) [46].

(1) $$Acid\;number\;(\mathrm{mg}\;KOH/g)=\frac{Volume\;of\;KOH\;used\;\left(\mathrm{mL}\right)\times Normality\;of\;KOH\times 56.1}{weight\;of\;polymer\;\left(g\right)}$$

(2) $$\mathrm{Degree}\;\mathrm{of}\;\mathrm{grafting}\;\left(\%\right)=\frac{Acid\;number\times 72}{2\times 56.1}\times 100\%$$

To observe the distribution of PAA in the nanofibers, a colorimetric method based on Toluidine Blue O (TBO) was used [47]. A 0.5 mM TBO solution with a pH of 10 was prepared in deionized water, and the nanofibers were added to this solution at 30 °C for 6 h. Any non-complex dyes adhering to the surface of the nanofibers were removed by washing them with deionized water. The dyed nanofibers were observed with an optical microscope.

### 2.5. Contact Angle Measurement

The dynamic contact angles of nanofibers were measured with a contact angle meter (CAM-121, Creating Nano, Tainan, Taiwan) under ambient conditions. The liquid selected for contact angle measurement was deionized water. Water droplet added to the surface of the nanofiber membrane, and the contact angle was recorded between 1 and 30 s.

### 2.6. Morphology and Characterization of Nanofibers

The surface morphology of nanofibers was examined using a scanning electron microscope (Hitachi S4100, Krefeld, Germany) operated at 3 kV. The magnification of the image was 3000 times. The nanofiber diameter was calculated from a group of 200 nanofibers, and the average diameter was determined. The nanofibers were characterized on the basis of the ^1^H-NMR spectrum with a Bruker-500 spectrometer, Billerica, MA, USA. The purified nanofibers were subjected to ^1^H-NMR spectrum analysis at 500 MHz with deuterochloroform (CDCl_3_) as the solvent.

### 2.7. Cell Viability

The experiment was carried out according to ISO 10993-5:2009 (*n* = 6). The detailed procedures for the metabolic activity of fibroblast NIH-3T3 cells cultured in the extract were described in previous studies [48]. A commercial AlamarBlueVR assay was used in measuring the effect of macrospheres on the metabolic activity of NIH-3T3 and identifying cell viability. The test was performed at intervals of 1 and 3 days after the initial seeding on the sample surface, and the samples were cultured in a 48-well culture plate in Dulbecco’s modified eagle medium at a density of 1 × 10^4^ cells per well. After the set time, the samples were cleaned twice with phosphate-buffered saline (PBS) and incubated in the culture medium for 4 h at 37 °C in a humidified 5% CO_2_ environment for the AlamarBlueVR assay. Then, an ELISA microplate reader (EZ Read 400, Biochrom, Cambridge, UK) was used to read the optical density at a wavelength of 490 nm. HDPE was used as the negative control group, and 15% DMSO was used as the positive control group.

### 2.8. Antimicrobial Ability Test

The antimicrobial ability of PCL/GO-*g*-PTA, and PCL-*g*-PAA-i1c1/GO-*g*-PTA nanofibers was evaluated with the viable cell count method. For the viable cell count method, a microbial solution with colony-forming units per milliliter (CFU/mL) ranging from 3 × 10^8^ to 3 × 10^10^ was prepared according to the dilution factor. The resulting microbial solution was incubated at 37 °C for 24 h. After incubation, 0.10 mL of the microbial solution was collected and diluted to 1 mL. When further dilution was required, the dilution procedure was repeated. The diluted solution was placed on an agar plate and further incubated for another 18 h. After incubation, the number of bacterial colonies in CFU/mL was calculated according to the number of cultured bacteria with an established protocol [49].

### 2.9. Biodegradation

Electrospun nanofibers were shaped into 1.5 cm × 1.5 cm. The samples were weighed and placed in 4 mL of PBS solution for the assessment of their biodegradation behavior. The weights of the samples were recorded in the first, second, and the third weeks, the fourth week, the sixth week, and the 60th day. Then, calculate the relative weight loss according to the following formula.

(3) $$\mathrm{Weight}\;\mathrm{reduction}\;(\%)=\frac{W_{0}-W_{i}}{W_{0}}\times 100\%$$

where *W*_0_ and *W_i_* were the sample weights before degradation and after the degradation, respectively.

## 3. Results and Discussion

### 3.1. Electrospun Nanofibers with the Mixture of PCL and AA

**Figure 2:**
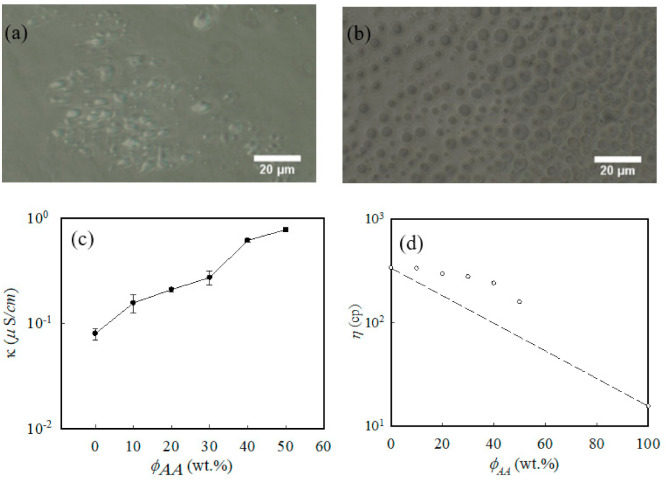
Optical microscopy (OM) photographs, conductivity, and viscosity of PCL/AA electrospinning solutions with different ratios of PCL and AA: (**a**) OM photograph of PCL/AA 80/20, (**b**) OM photograph of PCL/AA 50/50, (**c**) conductivity of the PCL/AA solutions, and (**d**) viscosity of the PCL/AA solutions.

The best ratio between PCL and AA in nanofibers for electrospinning was determined. Electrospinning solutions with different ratios of PCL and AA were studied. The composition of each solution is listed in Table 1. Before electrospinning, the pristine PCL solution was homogeneous. After AA was added, the solution became heterogeneous. A sphere-like domain appeared in the sample containing AA as shown in Figure 2a, and the phase separation became more obvious after the amount of AA was increased, as presented in Figure 2b.

The separation of the PCL and AA phases led to the heterogeneous behavior of the PCL/AA immiscible solution, and the heterogeneous behavior might have affected the morphology of the electrospun nanofibers. The effect is discussed in the subsequent section. Other factors that affected the morphology of the electrospun nanofibers were solution properties, such as viscosity and conductivity. Figure 2c shows the conductivity of the PCL/AA solutions with different ratios of AA and PCL. For the pristine PCL solution, the measured conductivity was approximately 0.09 ± 0.002 μS/cm. After 20 wt% AA was added to the PCL solution, the measured conductivity of the solution was approximately 0.40 ± 0.03 μS/cm, which was significantly higher than that of the pristine PCL solution. Given that AA generated ions in the solution, the conductivity of the solution increased with AA content. Figure 2d presents the viscosity of PCL/AA solutions with different AA and PCL ratios. As the amount of AA increased, the viscosity of the solution gradually decreased. AA is a low-viscosity monomer and not a polymer. A high-viscosity polymer mixed with a low-viscosity monomer changes the viscosity of a blend solution. However, the change did not follow the rule of the mixture and demonstrated a positive deviation, as shown in Figure 2d. The positive deviation of the viscosity observed in the PCL-rich blend solutions can be attributed to the strong interfacial interactions among phases in the solutions.

**Figure 3:**
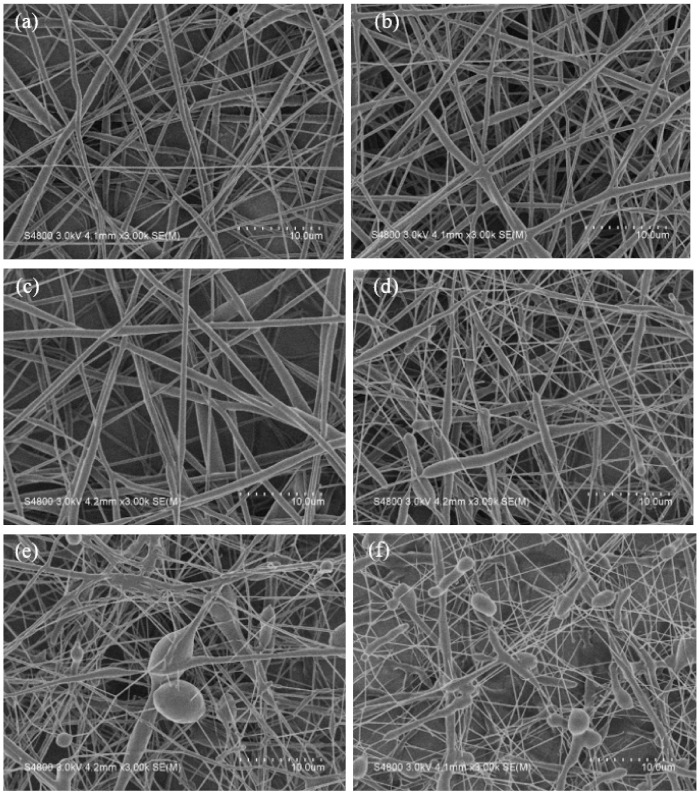
Scanning electron microscopy images of the electrospun nanofibers com-posed of different ratios of PCL and AA: (**a**) neat PCL, (**b**) PCL/AA 90/10, (**c**) PCL/AA 80/20, (**d**) PCL/AA 70/30, (**e**) PCL/AA 60/40, and (**f**) PCL/AA 50/50.

The effect of AA monomers on fiber morphology was investigated using electrospun nanofibers without photoinitiators and compatibilizers. Difference in morphology among the electrospun nanofibers was observed through SEM (Figure 3). 

As the amount of AA increased, the shape of the nanofibers changed from smooth (PCL/AA 80/20) to non-smooth (PCL/AA 70/30). When the amount of AA reached above 40 wt%, the beaded nanofibers were observed. The low viscosity and the excess amounts of AA caused the agglomeration of AA and led to the formation of the beaded nanofibers. The result implied that the immiscibility of the electrospinning solution affected the morphology of the electrospun nanofibers. The nanofiber morphology indicated that the best ratio of PCL/AA for the preparation of smooth electrospun PCL/AA nanofibers was 80/20. Therefore, the nanofiber with a PCL/AA ratio of 80/20 was further studied.

The average nanofiber diameter (*d_f_*) was obtained and listed in Table 1. The average diameter of PCL nanofiber was 780 ± 200 nm. As the composition of AA increased, the average diameters of nanofibers decreased. The average diameters of PCL/AA were 566 ± 261, 557 ± 271, 497 ± 361, 363 ± 256, and 330 ± 321 nm in 90/10, 80/20, 70/30, 60/40, and 50/50, respectively. In electrospinning technology, solution viscosity and conductivity are important factors that determine the diameter of an electrospun fiber. In previous studies [50,51], *d_f_* decreased as the viscosity of the solution decreased and the conductivity of the solution increased. The addition of AA to PCL reduced the viscosity of the solution and increased the conductivity of the solution, as shown in Figure 2. Therefore, changes in the average diameters of the nanofibers with AA were consistent with the trends previously reported [50,51]. In addition to the influence of the conductivity and viscosity of the solutions, another reason for the decrease in the average diameter was the increase in the number of fine fibers with AA content increased. The result implied that PCL rather than AA was the backbone of the nanofiber.

### 3.2. Electrospun Nanofibers with PCL Grafted with PAA

For the grafting of AA onto PCL, the compatibilizer and photoinitiator were added to the PCL/AA solution. The compatibilizer and photoinitiator played different roles in the synthesis. Speedcure 73 produced free radicals and reacted with PCL, AA, and EDGMA. By contrast, EDGMA reacted mainly with AA monomers and PCL and facilitated the grafting of AA graft onto PCL. By the illumination of the electrospinning process, the photoinitiator generated free radicals. The free radicals produced by the photoinitiator reacted with the methylene segments of the PCL molecular chain, facilitating the grafting of AA monomers onto the surface of PCL and formation of PCL-*g*-PAA nanofibers. PCL/AA 80/20 nanofibers with various amounts of the compatibilizer and photoinitiator were prepared and used in exploring the effects of different ratios of the compatibilizer and photoinitiator in the PCL/AA solution during electrospinning. The combinations of different proportions of compatibilizer and photoinitiator were 0, 1, 5, and 10 wt% according to the amount of AA monomers.

**Figure 4:**
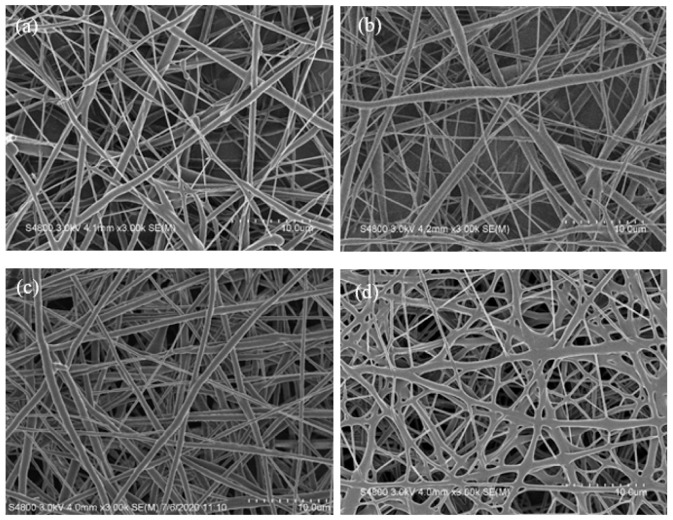
SEM images of the PCL-*g*-PAA electrospun nanofibers: (**a**) PCL-*g*-PAA-i1, (**b**) PCL-*g*-PAA-i10, (**c**) PCL-*g*-PAA-i1c1, and (**d**) PCL-*g*-PAA-i1c10.

Photoinitiator (1 wt%) was added to the solution. The SEM image showed rough electrospun nanofibers (Figure 4a). In each nanofiber, the diameter varied along the fiber direction. The distribution of the electrospun nanofiber diameter demonstrated two peaks. After the photoinitiator content increased to 10 wt%, the electrospun nanofiber presented further nonsmooth and showed a beaded fiber, as shown in Figure 4b. When the content of the photoinitiator was maintained at 1 wt%, the presence of 1 wt% compatibilizer smoothed the electrospun nanofiber (Figure 4c). When the content of the compatibilizer reached 10 wt%, the gluing between cross-linked nanofibers was observed (Figure 4d).

**Figure 5:**
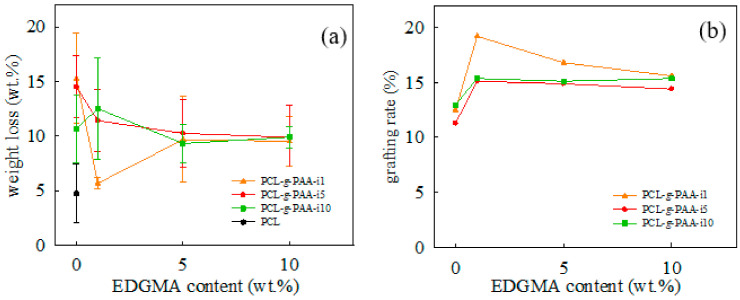
Weight loss percentage and grafting rate of the PCL-*g*-PAA electrospun nanofibers: (**a**) weight loss percentage during purification; (**b**) grafting rate obtained through acid–base titration.

Figure 5 shows the weight loss percentage and grafting rate of the twelve different nanofibers after purification. The purpose of purification was to remove AA or PAA short chains that did not attach to the PCL backbones through chemical bonds during electrospinning with in situ photopolymerization. The weight loss percentage of the PCL-*g*-PAA during the purification was recorded in Figure 5a. The grafting rate of the purified PCL-*g*-PAA was obtained with the acid-base titration test conducted on nanofibers. The results are presented in Figure 5b. Weight loss percentage and grafting rate can be used as the indicators of the result of grafting of PAA onto PCL.

Regarding the weight loss percentage shown in Figure 5a, the pristine PCL nanofiber showed a weight loss of approximately 4.72 ± 2.70 wt%. The 5% weight loss can be treated as the background loss for the purification and functioned as the control group. Therefore, the highest possible weight loss for electrospinning with in situ photopolymerization was 25 wt% (20 wt% of unreacted AA monomers and the 5 wt% background weight loss). Without the compatibilizer, the addition of 1 wt% photoinitiator resulted in the highest weight loss of approximately 15.28 ± 4.15 wt% during electrospinning. As the content of the photoinitiator reached 10 wt%, the weight loss decreased to 10.67 ± 3.13 wt%. This result implied that the photoinitiator can facilitate the PAA grafting from the PCL chain. After the addition of 1 wt% compatibilizer, the nanofiber with 1 wt% photoinitiator had the lowest weight loss percentage of approximately 5.66 ± 0.53 wt%, which was close to that in the pristine PCL nanofiber. However, when the content of photoinitiators increased, the weight loss percentage increased as well. At 5 wt% of the photoinitiator, as the content of the compatibilizer increased, the weight loss percentage decreased, and the compatibilizer reached saturation at about 1 wt%. The weight loss percentage was slightly higher in the 5 wt% of the photoinitiator than in the 1 wt%. When the content of the photoinitiator was 10 wt%, the compatibilizer increased from 1 wt% to 10 wt%, the weight loss percentage fluctuated slightly, but all values were within the error range of the weight loss percentage of the sample without the compatibilizer. However, nanofibers with 10 wt% photoinitiator demonstrated a higher weight loss percentage than the pristine PCL nanofibers.

As shown in Figure 5b, grafting rate was analyzed using the acid–base titration test. Without the compatibilizer, the grafting rate of the nanofibers with 1 wt% photoinitiator was 12.48%. When the content of the photoinitiator increased to 5 wt%, the grafting rate dropped slightly to 11.28%. Photoinitiator content further increased to 10 wt%, and the grafting rate increased to 12.96%. The results showed that the content of the photoinitiator had little effect on the grafting of PAA onto PCL, and the grafting rate was approximately 11–12%. In the absence of the compatibilizer, the presence of the photoinitiator initiated the grafting reaction but did not promote the grafting rate. When photoinitiator content was 1 wt%, the highest grafting rate of approximately 19.20% was obtained after the addition of 1 wt% compatibilizer. However, as the content of the compatibilizer further increased, the grafting rate decreased. When the content of the photoinitiator was 5 or 10 wt%, the trend observed after the addition of the compatibilizer was similar to that observed in nanofibers with 1 wt% photoinitiator. The results showed that the heterogeneous phase between hydrophilic AA and hydrophobic EGDMA promoted the reaction of the additional compatibilizer reacted with itself and thereby decreased the grafting rate. The compatibilizer reached saturation at approximately 1 wt%.

Weight loss percentage and grafting rate presented that the formula used and the in situ polymerization during electrospinning resulted in the successful grafting of PAA onto the PCL molecular chain. Given that the AA monomer and PCL are immiscible, AA monomers were not completed grafted on the PCL molecule when the photoinitiator was used alone. The molecular structure of the compatibilizer had two ester groups that are compatible with the ester group of PCL and the carboxylic acid groups of the AA monomers, and the compatibilizer acted as a bridge connecting PCL to AA monomers. Therefore, a small amount of EDGMA facilitated the reaction of the AA monomers on the PCL surface and increased the grafting rate, but further increase in EDGMA did not increase the number of AA monomers grafted onto PCL. As shown in Figure 5a,b, the lowest weight loss percentage and highest grafting rate were obtained when 1 wt% compatibilizer and 1 wt% photoinitiator were used. Excessive amounts of the compatibilizer and photoinitiators did not facilitate the grafting of PAA onto PCL. The compatibilizer contributed to the formation of the hydrogel PAA, which promoted the grafting rate. Therefore, PCL/AA-(80/20) with 1 wt% compatibilizer and 1 wt% photoinitiator was the best composition ratio and denoted as PCL-*g*-PAA-i1-c1.

Figure S2 showed the FTIR spectra of the electrospun PCL, PCL/AA 80/20, and PCL-*g*-PAA-i1c1 nanofibers. Similar FTIR results were obtained in the electrospun PCL, PCL/AA 80/20, and PCL-*g*-PAA-i1c1 nanofibers. The absorbance bands at 3000–2800 cm^−1^ were detected with the asymmetric stretching of the methylene groups (C–H of CH_2_). In addition, the absorbance bands at 1725 and 1180 cm^−1^ were assigned to C=O and C-O groups, respectively. In the electrospun PCL/AA 80/20 and PCL-*g*-PAA-i1c1 nanofibers, the new broad peak at approximately 3600~3000 cm^−1^ was attributed to the O–H stretching vibration of the acrylic acid group. The purified powder of the electrospun PCL-*g*-PAA-i1c1 nanofibers was subjected to ^1^H-NMR analysis. The formation of PCL-*g*-PAA chemical structure was confirmed using the ^1^H-NMR spectrum, and the result is shown in Figure S3. The strong methylene groups of characteristic PCL peaks (denoted as a–d) were observed at *δ* values of 4.06, 2.31, 1.65, and 1.38 ppm. The peaks for methine proton (–CH–) in the PAA main chain appeared at 1.7 and 2.2 ppm [52]. The absence of the proton peaks δ = 6–7) of –CH=CH– in the AA monomers indicated the absence of AA monomers. Notably, the nanofibers were purified, and thus all the functional groups originated from PAA rather than from AA monomers. However, the presence of small peaks implied that PAA was in the oligomer form instead of the long-chain polymer form. The ^1^H-NMR spectra showed the formation of PAA, and the segment of PAA was grafted from PCL.

**Figure 6:**
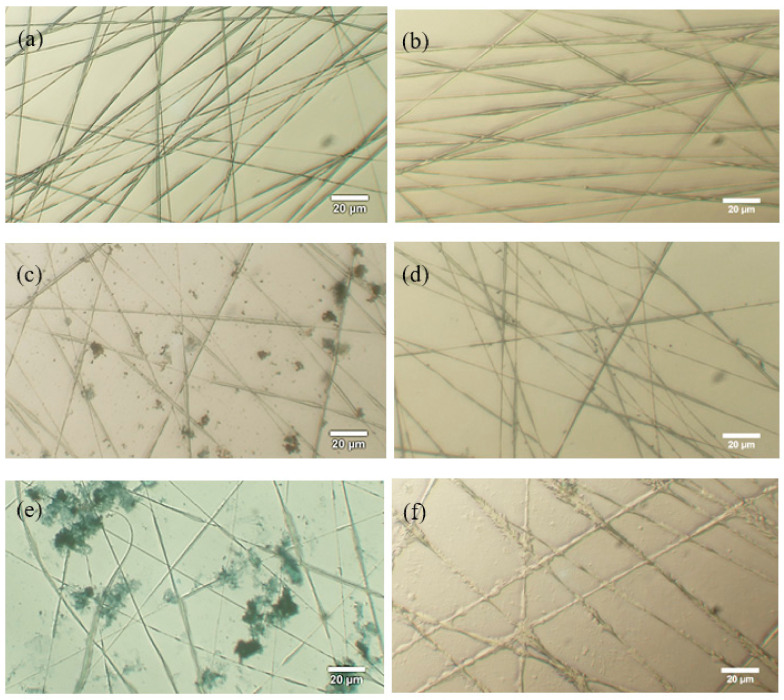
OM images of different electrospun nanofibers dyed with TBO dye solution and TBO-dyed electrospun nanofibers washed with NaOH solution: (**a**,**c**,**e**) different electrospun nanofibers dyed with TBO dye solution; (**a**) neat PCL, (**c**) PCL-*g*-PAA-i1, (**e**) PCL-*g*-PAA-i1c1; (**b**,**d**,**f**) TBO dying electrospun nanofibers washed with NaOH solution; (**b**) neat PCL, (**d**) PCL-*g*-PAA-i1, (**f**) PCL-*g*-PAA-i1c1.

Figure 6a,c,e present the optical microscopy (OM) images of different electrospun nanofibers dyed with TBO dye solution. Given that the amine group of TBO combined with the acid group of AA or PAA and determined the location of AA or PAA, the morphological distribution of the electrospun nanofiber was observed through dyeing. As shown in Figure 6a, the dye completely disappeared in the pristine PCL electrospun nanofiber because of the hydrophobic nature of PCL. As shown in Figure 6c, PCL-*g*-PAA-i1 was the nanofiber made with 1 wt% photoinitiator. The dye molecules were near the nanofiber, and few dye molecules attached to the fiber, implying that the AA monomers did not undergo chemical bonding with PCL and few PAA molecules attached to the surfaces of the PCL nanofibers. The dye molecules aggregated and attached to the electrospun nanofibers with 1 wt% photoinitiator and 1 wt% compatibilizer (Figure 6e). The presence of the photoinitiator and compatibilizer initiated the grafting of PAA onto PCL. Moreover, PAA was mainly grafted onto the surfaces of the fibers. Figure 6b,d,f show the OM images of TBO-dyed electrospun nanofibers washed with a NaOH solution. Figure 6b and d present the NaOH-washed nanofibers of the pristine PCL and PCL-*g*-PAA-i1. Both nanofibers demonstrated smooth fiber surfaces. By contrast, the PCL-*g*-PAA-i1c1 nanofibers (Figure 6f) manifested roughly brushed surfaces, which were caused by the removal of PAA through a reaction with NaOH. The OM images indicated that more PAA was grafted from the PCL nanofibers in the presence of the compatibilizer. The result was compatible with the result of weight loss percentage and grafting rate. PCL-*g*-PAA-i1c1 had the optimal composition for in situ polymerization during electrospinning. The result demonstrated the non-uniform core/shell morphology of the electrospun PCL-*g*-PAA nanofibers, in which PCL was the core and PAA was the shell.

When the PCL/AA phase separation solution was subjected to electrospinning, the small molecule AA had a low viscosity and high conductivity and moved to the surface of the liquid through electrostatic force after the solution exited the electro-spinneret. At this time, the photopolymerization reaction started because of the exposure of the electrospinning solution to light and that PAA was grafted from the surface of the PCL nanofiber. The compatibilizer acted as a bridge between PCL and PAA and facilitated the formation of PCL-*g*-PAA nanofibers with core-shell structures through the electrospinning with the in situ polymerization (Figure 1).

The membranes made with PCL and PCL-*g*-PAA nanofibers were subjected to water contact angle testing. The result is shown in Table 1. The membrane made of the pristine PCL nanofiber demonstrated its hydrophobic property and had a water contact angle of 132°. It did not absorb water as the water absorption time was not available. The membranes made with PCL/AA and PCL-*g*-PAA nanofibers presented a water absorption time of 35 s or less. Because of the high porosity of the nanofiber mats, water would be absorbed by the nanofiber mat, which was changed to hydrophilicity. Thus, the water contact angles were listed as 0 for all PCL/AA and PCL-g-PAA membranes in Table 1. Grafting and polymerization did not prevent PAA from improving the hydrophobic property of PCL.

### 3.3. Biodegradation of Electrospun PCL and PCL-g-PAA Nanofibers

**Figure 7:**
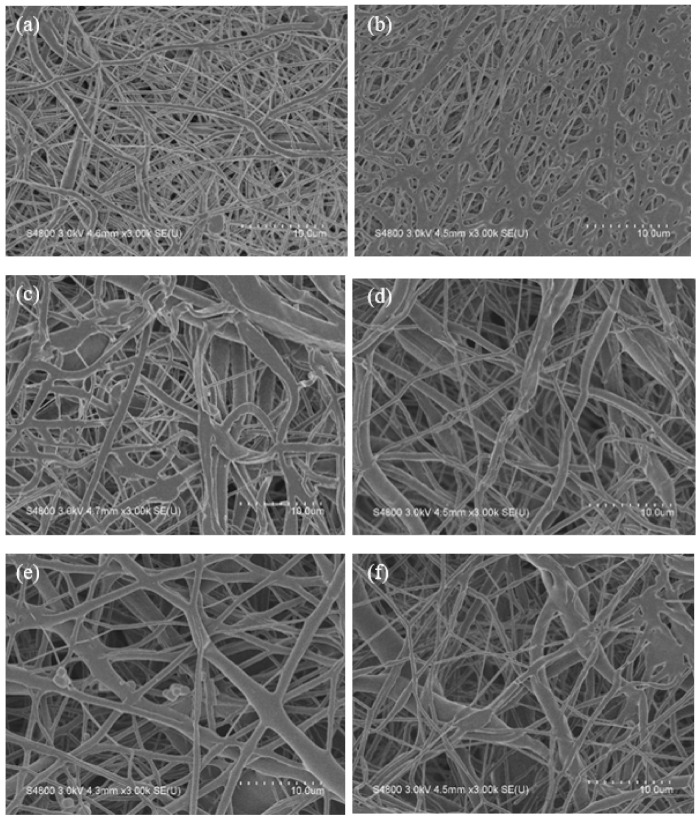
SEM images of biodegraded PCL, PCL-*g*-PAA-i1, and PCL-*g*-PAA-i1c1 nanofiber mats in PBS solution for 42 and 60 days. At 42 days, (**a**) neat PCL, (**c**) PCL-*g*-PAA-i1, (**e**) PCL-*g*-PAA-i1c1; At 60 days, (**b**) neat PCL, (**d**) PCL-*g*-PAA-i1, (**f**) PCL-*g*-PAA-i1c1.

PCL is an essentially biodegradable polymer and mainly degraded through the hydrolysis of ester bonds on its molecular chains. This process generates carboxylic acid and hydroxyl functional groups, which in turn reduces the molecular weight and finally decreases the overall weight of the polymer. The biodegradation rate of PCL is related to molecular weight, molecular weight distribution, crystallinity, and hydrophobicity of PCL molecular chains [53]. Figure 7 shows the SEM images of biodegraded PCL, PCL-*g*-PAA-i1, and PCL-*g*-PAA-i1c1 nanofiber mats in PBS solution for 42 and 60 days.

All nanofiber mats demonstrated changes in morphology compared with the non-biodegradation mats, as shown in Figure 3a and Figure 4a,c. In the PCL nanofiber mat, the 42-day sample had curved and round nanofibers (Figure 7a). The 60-day sample had a flat ribbon-like nanofiber although the layout of the nanofiber did not show obvious difference (Figure 7b). In the PCL-*g*-PAA-i1 nanofiber mat, the 42- and 60-day samples presented large biodegradation areas, which were not observed in the PCL nanofiber mat. In the 42-day sample, cracks appeared on the nanofibers. These cracks were caused by the degradation of the nanofibers, as shown in Figure 7c. In the 60-day sample, breaks were found in the nanofibers, except in the cracks, as shown in Figure 7d. In the PCL-*g*-PAA-i1c1 nanofiber mat, the 42- and 60-day samples displayed fractures in the nanofibers (Figure 7e,f). In short, severe biodegradation occurred in the 60-day samples compared with the 42-day samples.

**Figure 8:**
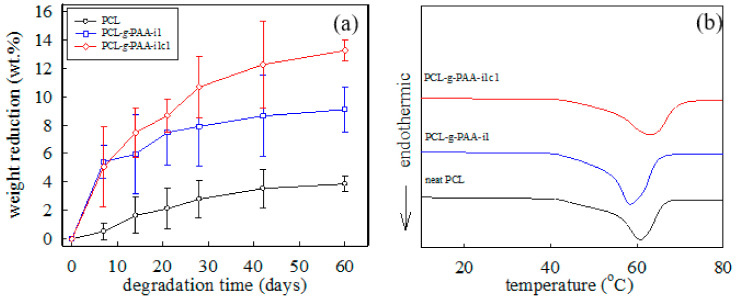
Dependence of biodegradation time on weight reduction (%) and DSC heating traces of the PCL, PCL-*g*-PAA-i1, and PCL-*g*-PAA-i1c1 nanofibers: (**a**) dependence of the biodegradation time on weight reduction (%); (**b**) DSC heating traces of the electrospun nanofibers.

Figure 8a demonstrates that biodegradation time depended on the weight reduction (%) of the PCL nanofiber, PCL-*g*-PAA-i1 nanofiber, and the PCL-*g*-PAA-i1c1 nanofiber. The presence of PAA increased the weight reduction rates of the nanofibers from 4 wt% to 13 wt% on the 60th day while comparing PCL-*g*-PAA-i1c1 nanofibers with pure PCL nanofibers. When comparing PCL-*g*-PAA-i1 and PCL-*g*-PAA-i1c1, the addition of compatibilizer also increased the weight reduction of nanofibers on the 60th day from 8 wt% to 13 wt%. The results showed that EDGMA increased the PAA grafting rate and led to a higher weight loss. The biodegradation results indicated that PCL is more resistant to biodegradation than PCL-*g*-PAA. Therefore, the grafting of PAA on PCL promoted the biodegradation of the nanofibers.

Figure 8b shows the DSC heating traces of the electrospun PCL, PCL-*g*-PAA-i1, and PCL-*g*-PAA-i1c1 nanofibers. Melting peak temperature and endothermic enthalpy were denoted as *T_m_* and Δ *H_m_*, respectively. The *T_m_* of neat PCL nanofibers was 60.8 °C. The *T_m_* of the PCL-*g*-PAA-i1 nanofibers slightly shifted to 58.5 °C. In the electrospun nanofibers made with 1 wt% photoinitiator and 1 wt% compatibilizer, *T_m_* increased to 63.1 °C. Melting point is related to the lamella thickness. Based on the results of the weight loss and grafting rate, the amount of the unreacted AA monomers in the electrospun PCL-*g*-PAA-i1 nanofibers were more than that in the electrospun PCL-*g*-PAA-i1c1 nanofibers. The presence of unreacted AA monomers might retard PCL crystallization. Therefore, a high content of unreacted AA monomers yielded electrospun PCL nanofibers with thin lamellae. The presence of compatibilizer promoted the grafting of PAA from the PCL and yielded electrospun PCL nanofibers with thick lamellae. PCL-*g*-PAA-i1c1 nanofibers had the highest *T_m_*.

For the comparison of the amounts of melting crystals, Δ*H_m_* was normalized with the PCL content for the derivation of Δ*H’_m_* = [Δ*H_m_*/(*1–ϕ_AA_*)]. After the addition of 20 wt% AA content, the Δ*H’_m_* of the PCL nanofiber significantly increased from 62.8 J/g for the neat PCL nanofiber to 83.4 J/g and 77.9 J/g for the PCL-*g*-PAA-i1 nanofibers and the PCL-*g*-PAA-i1c1 nanofibers, respectively. In addition, the crystallinity fraction (*ϕ^DSC^*) was calculated using the expression Δ*H’_m_*/Δ$H_{m}^{o}$, where Δ$H_{m}^{o}$ = 139.5 J/g, which is the melting enthalpy of 100% pure PCL crystals [54]. The derived *ϕ^DSC^* values for electrospun PCL, PCL-*g*-PAA-i1, and PCL-*g*-PAA-i1c1 nanofibers were 0.45, 0.60, and 0.56, respectively. This result indicated that PCL nanofibers with grafted PAA increased the degree of crystallinity during electrospinning. The biodegradation of the PCL began from the amorphous segments. The electrospun PCL-*g*-PAA nanofiber mats exhibited a high degree of crystallinity but a severe biodegradation rate. This phenomenon implied that the crystallinity was not the main factor in biodegradation. However, hydrophilicity was the dominant factor in PCL biodegradation. The presence of PAA in PCL improved the hydrophilicity of the PCL and promoted its biodegradation.

### 3.4. Filled PCL and PCL-g-PAA Nanofibers with GO-g-PTA Nanosheets

**Figure 9:**
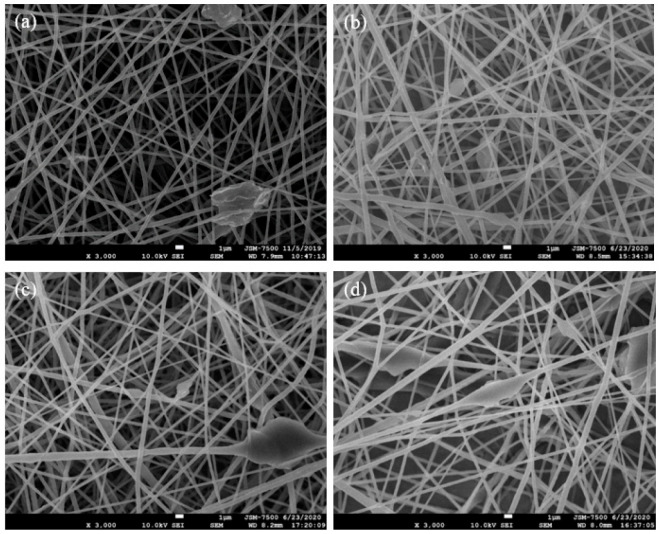
SEM images of composite membranes composed of different proportions of GO-g-PTA nanosheets filled with PCL and PCL-g-PAA nanofibers: (**a**) PCL/GO-g-PTA 95/5, (**b**) PCL-g-PAA-i1c1/GO-g-PTA 95/5, (**c**) PCL/ GO-g-PTA 90/10, and (**d**) PCL-g-PAA-i1c1/GO-g-PTA 90/10.

After electrospinning and in situ photopolymerization, PAA improved the hydrophilicity and biodegradation of the PCL nanofibers. In this study, GO-*g*-PTA, which has excellent antibacterial properties and biocompatibility, was added to electrospun PCL and PCL-*g*-PAA composite nanofibers, and the effects of filling PCL and PCL-*g*-PAA nanofibers with GO-*g*-PTA on fiber morphology, antibacterial properties, and cell viability were investigated. PCL-*g*-PAA-i1c1 was selected for further study because it had the highest PAA grafting rate. Figure 9 displays the SEM images of the composite membranes composed of different contents of GO-*g*-PTA nanosheets filled with PCL and PCL-*g*-PAA nanofibers.

As shown in the SEM images in Figure 9, the PCL and PCL-*g*-PAA composite nanofibers became less smooth and formed irregular protruded structures along the fibers. The irregular protruded structures, which increased with GO-*g*-PTA content, indicated the position of the GO-*g*-PTA nanofillers. The lateral dimension of GO-*g*-PTA was larger than the diameters of the electrospun PCL and PCL-*g*-PAA nanofibers, and the GO-*g*-PTA particles protruded from the smooth electrospun nanofiber, as demonstrated in Figure S4. Even when the amount of GO-*g*-PTA nanosheets reached 10 wt%, the electrospun nanofibers were successfully prepared, as shown in Figure 9c,d. No difference was observed between the PCL and PCL-*g*-PAA composite mats. The morphology of GO-*g*-PTA in the PCL-based electrospun nanofibers was consistent with that of PVA/GO-*g*-PTA composite mats [44].

The calculated *d_f_* values are listed in Table 2. The average diameter of the PCL nanofiber was 780 ± 200 nm, and the average diameter of the PCL-g-PAA nanofiber was 618 ± 212 nm. After the addition of GO-g-PTA nanosheets to the PCL and PCL-g-PAA nanofibers, the average diameter of nanofibers was approximately 400 nm for all composition ratios. The filling of GO-g-PTA nanosheets reduced the average fiber diameter of the electrospun nanofibers. The lowest average fiber diameter was obtained after the addition of 3 wt% GO-g-PTA, and the lowest value resulted from the trade-off between high viscosity and high electric conductivity in the presence of GO-g-PTA. In previous studies [50,51], the high viscosity of the electrospinning solution increased *d_f_*, and the high solution conductivity decreased *d_f_*. The competition between the two factors accounted for the lowest *d_f_* of the electrospun nanofibers.

The results of the water contact angle test on the composite mats are shown in Table 2. The composite membranes made with PCL nanofibers, such as PCL/GO-*g*-PTA 95/5 and PCL/GO-*g*-PTA 90/10, showed the hydrophobic property, and the membranes did not absorb water (Table 2). However, the composite membranes made with PCL-*g*-PAA nanofibers, such as PCL-*g*-PAA/GO-*g*-PTA 95/5 and PCL-*g*-PAA/GO-*g*-PTA 90/10, presented not only the ability to absorb water but also had a water contact angle comparable to that of a PCL-*g*-PAA membrane (Table 2). The composite mats made with PCL-*g*-PAA nanofibers filled with GO-*g*-PTA nanofillers demonstrated a hydrophilic property because of the presence of PAA.

### 3.5. Antimicrobial Ability and Cell Viability Tests

**Figure 10:**
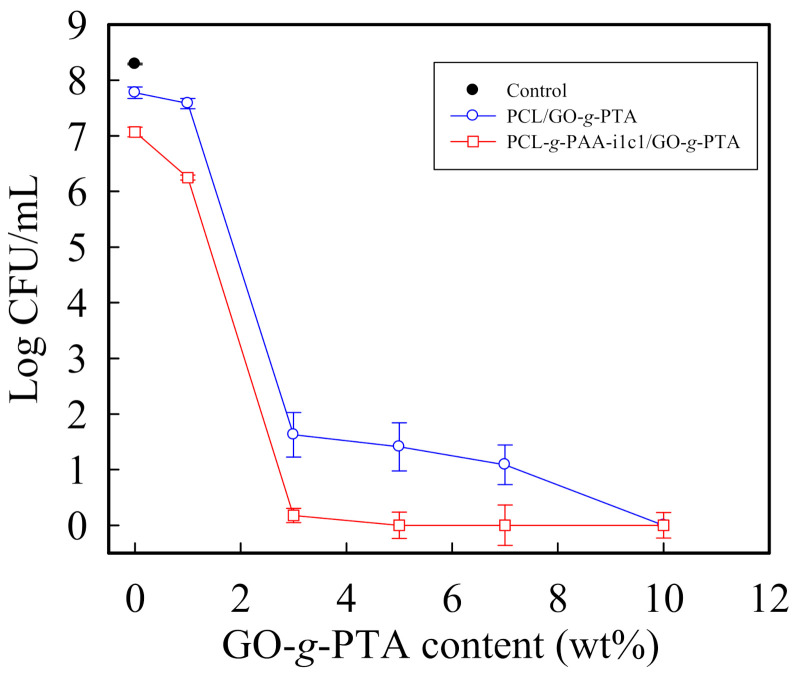
Log (CFU/mL) versus the concentration of PCL/GO-*g*-PTA and PCL-*g*-PAA-i1c1/GO-*g*-PTA composite mats against *S. aureus*.

The antibacterial activities of the PCL/GO-*g*-PTA and PCL-*g*-PAA-i1c1/GO-*g*-PTA composite mats were evaluated. The viable cell count method was used in evaluating their antibacterial activities against *Staphylococcus aureus* through a broth microdilution assay [55]. The minimal bactericidal concentration (MBC) in this section is the minimum concentration of tested biocidal agents or fillers used to fill PCL and PCL-*g*-PAA-i1c1 composite nanofiber mats in which 99.9% of the initial bacterial colony is killed [49]. Figure 10 shows the relationship between the number of bacterial colonies in CFU/mL and the concentrations of PCL/GO-*g*-PTA and PCL-*g*-PAA-i1c1/GO-*g*-PTA composite mats. The number of bacterial colonies in was 5.96 × 10^7^ CFU/mL in the neat PCL, and 1.17 × 10^7^ CFU/mL in the neat PCL-*g*-PAA-i1c1 nanofiber mat. All bacterial colonies were completely killed by either the PCL/GO-*g*-PTA composite mat with 10 wt% GO-*g*-PTA or PCL-*g*-PAA-i1c1/GO-*g*-PTA composite mat with 5 wt% GO-*g*-PTA. The MBC wt% value of the PCL/GO-*g*-PTA composite nanofiber mat was higher than that of the PCL-*g*-PAA-i1c1/GO-*g*-PTA composite nanofiber mat. Therefore, the PCL-*g*-PAA-i1c1/GO-*g*-PTA composite nanofiber mat had better antimicrobial ability against *S. aureus* than the PCL/GO-*g*-PTA composite nanofiber mat. The antibacterial activity results showed that the hydrophilic PCL-*g*-PAA/GO-*g*-PTA composite mat exhibited more effective antibacterial activity than the hydrophobic PCL/GO-*g*-PTA composite mat. The MBCs of PVA/GO-*g*-PTA and PCL/GO-*g*-PTA composite mats were comparable, and the MBC had a value of 10 wt% GO-*g*-PTA [44]. The antimicrobial abilities of the electrospun PVA and PCL nanofibers filled with GO-*g*-PTA nanosheets were the same. However, the MBC of the PCL-*g*-PAA/GO-*g*-PTA composite mats was lower than that of PVA/GO-*g*-PTA. Therefore, the antimicrobial ability of the PCL-*g*-PAA/GO-*g*-PTA composite mats was better than that of PVA/GO-*g*-PTA. In this study, the in situ photopolymerization of PAA grafted onto the electrospun PCL nanofibers resulted in the formation of a core-shell fiber structure and improved the hydrophilicity of PCL. The synergy of the hydrophilicity of the PCL-*g*-PAA nanofibers and the antimicrobial ability of the GO-*g*-PTA nanosheets decreased the MBC of the composite mat and implied enhanced antimicrobial ability.

**Figure 11:**
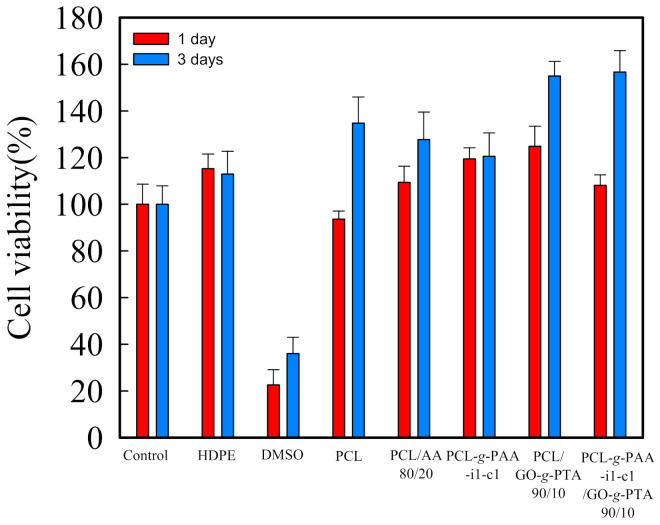
Cell viability of the treated NIH-3T3 cells for the neat PCL, PCL/AA 80/20, PCL-*g*-PAA-i1c1, PCL/GO-*g*-PTA 90/10, and PCL-*g*-PTA-i1c1/GO-*g*-PTA composite nanofiber mats after 1 and 3 days of incubation.

Moreover, fibroblast NIH-3T3 cells were selected as model cells for evaluating the cell viability of the PCL composite nanofiber mats. The nanofiber mats made with PCL, PCL/AA-(80/20), PCL-*g*-PAA, PCL/GO-*g*-PTA, and PCL-*g*-PAA/GO-*g*-PTA nanofibers were tested for in vitro cytotoxicity after 1 and 3 days of incubation. The results are presented in Figure 11. HDPE was the negative control group, and DMSO was the positive control group. When the mats were toxic, the data presented would be lower than 80%. No significant difference in cell viability was found among the composite mats, and all mats exhibited characteristics close to those of HDPE. Notably, the presence of 20 wt% AA monomers or PAA in the composite mats did not result in cytotoxicity. The cell viability test showed that all composite mats prepared in this study had no cytotoxicity under experimental control conditions.

## 4. Conclusions

The optimization of in situ polymerization for the preparation of composite nanofiber mats used in chronic wound care was studied. The mats were composed of PCL-*g*-PAA and GO-*g*-PTA. We demonstrated the PCL-*g*-PAA nanofibers with core shell structures can be prepared through electrospinning with in situ photopolymerization. The best composition ratio for preparing PCL-*g*-PAA was PCL/AA 80/20 with 1 wt% compatibilizer and 1 wt% photoinitiator. Moreover, PCL-*g*-PAA-i1c1/GO-*g*-PTA 97/3 was the most suitable nanofiber for chronic wound mat applications. Polymerization did not prevent PAA from modifying the hydrophobic property of PCL; therefore, the composite membranes made with PCL-*g*-PAA nanofibers improved the hydrophobic property of PCL. Change in hydrophobicity promoted the biodegradation of the composite membrane, and the cell viability and antibacterial activity tests for the composite nanofiber mat verified that the mat had no cytotoxicity and had effective antibacterial properties. Our findings may provide important information regarding the in situ polymerization for various potential applications of electrospun PCL-*g*-PAA and GO-*g*-PTA nanofibers used as polymer-based biomaterials for chronic wound management.

## Figures and Tables

**Table 1:** Composition and the average diameter of electrospun PCL and AA nanofibers.

Sample Code	PCL (wt%)	AA (wt%)	Photoinitiator (wt%)	EDGMA (wt%)	*d_f_* (nm)	Water Contact Angle (°)
PCL/AA 100/0	100	0	-	-	780 ± 200	132
PCL/AA 90/10	90	10	-	-	566 ± 261	0
PCL/AA 80/20	80	20	-	-	557 ± 271	0
PCL/AA 70/30	70	30	-	-	497 ± 361	0
PCL/AA 60/40	60	40	-	-	363 ± 256	0
PCL/AA 50/50	50	50	-	-	330 ± 321	0
PCL-g-PAA-i1	80	20	1	-	660 ± 301	0
PCL-*g*-PAA-i5	80	20	5	-	602 ± 273	0
PCL-*g*-PAA-i10	80	20	10	-	591 ± 304	0
PCL-*g*-PAA-i1c1	80	20	1	1	618 ± 212	0
PCL-*g*-PAA-i1c10	80	20	1	10	513 ± 203	0

**Table 2:** Composition, average diameter, and water contact angle of electrospun PCL/GO-*g*-PTA and PCL-*g*-PAA-i1c1/GO-*g*-PTA composite nanofiber dressings.

Sample Code	PCL (wt%)	PCL-*g*-PAA-i1c1 (wt%)	GO-*g*-PTA (wt%)	*d_f_* (nm)	Water Contact Angle (°)
PCL/GO-*g*-PTA 99/1	99	-	1	443 ± 130	134
PCL/GO-*g*-PTA 97/3	97	-	3	414 ± 122	131
PCL/GO-*g*-PTA 95/5	95	-	5	422 ± 102	127
PCL/GO-*g*-PTA 93/7	93	-	7	481 ± 128	128
PCL/GO-*g*-PTA 90/10	90	-	10	438 ± 122	126
PCL-*g*-PAA-i1c1/GO-*g*-PTA 99/1	-	99	1	499 ± 186	0
PCL-*g*-PAA-i1c1/GO-*g*-PTA 97/3	-	97	3	353 ± 94	0
PCL-*g*-PAA-i1c1/GO-*g*-PTA 95/5	-	95	5	520 ± 204	0
PCL-*g-*PAA-i1c1/GO-*g*-PTA 93/7	-	93	7	490 ± 202	0
PCL-*g*-PAA-i1c1/GO-*g*-PTA 90/10	-	90	10	431 ± 138	0

## Supplementary Materials

The following are available online at https://www.mdpi.com/article/10.3390/polym13234246/s1, Figure S1. Functional domain for the electrospinning solutions of 14 wt% PCL with (a) various AA contents, (b) various GO-*g*-PTA contents, and (c) 20 wt% AA and various GO-*g*-PTA contents. The domains indicated the range of operating electrical fields required for the stable cone-jet mode. (Filled symbols for lower bond applied voltage and open symbols for upper bond applied voltage). Figure S2. The FTIR spectra of the electrospun PCL, PCL/AA 80/20, and PCL-*g*-PAA-i1c1 nanofibers. Figure S3. ^1^H-NMR spectra of PCL-*g*-PAA. Figure S4. TEM images of electrospun PCL and PCL-*g*-PAA composite nanofibers filled with various amounts of GO-g-PTA. (a) PCL/GO-*g*-PTA 95/5, (b) PCL-*g*-PAA-i1c1/GO-*g*-PTA 95/5, (c) PCL/ GO-*g*-PTA 90/10, and (d) PCL-*g*-PAA-i1c1/GO-*g*-PTA 90/10.
