# Supplementary material for: Microstructure and Biological Properties of Electrospun In Situ Polymerization of Polycaprolactone-Graft-Polyacrylic Acid Nanofibers and Its Composite Nanofiber Dressings
Source: Polymers (Basel). 2021 Dec 3;13(23):4246. doi: 10.3390/polym13234246 (PMC8659835; doi:10.3390/polym13234246)
Supplement: Supplementary file 1 [file polymers-13-04246-s001.zip › polymers-1452585-supplementary.pdf]

Supplementary Material

Microstructure and Biological Properties of Electrospun In-situ Polymerization of Polycaprolactone-Graft-Polyacrylic Acid Nanofibers and Its Composite Nanofiber Dressings

Yi-Jen Huang [1,‡], Chien-Lin Huang [1,‡,*] Ruo-Yu Lai [1], Cheng-Han Zhuang [1], Wei-Hao Chiu [2], and Kun-Mu Lee [2,3,4,*]

[1] Department of Fiber and Composite Materials, Feng Chia University, Taichung 40724, Taiwan; yijenhua@gmail.com (Y.-J.H.); clhuang@mail.fcu.edu.tw (C.-J.H.); judy91206@gmail.com (R.-Y.L.); skyforest1316@gmail.com (C.-H.Z.)

[2] Center for Green Technology, Chang Gung University, Taoyuan 33302, Taiwan; kmlee@mail.cgu.edu.tw (K.-M.L.); weihao.chiu@gmail.com (W.-H.C.)

[3] Department of Chemical and Materials Engineering, Chang Gung University, Taoyuan 33302, Taiwan

[4] Department of Pediatrics, Chang Gung Memorial Hospital, Linkou, Taoyuan 33305, Taiwan

‡ These authors contributed equally to this work

* Correspondence: clhuang@mail.fcu.edu.tw(C.-J.H.); kmlee@mail.cgu.edu.tw (K.-M.L.); Tel.: +886-4-24517250 (ext. 3437) (C.-L.H.)

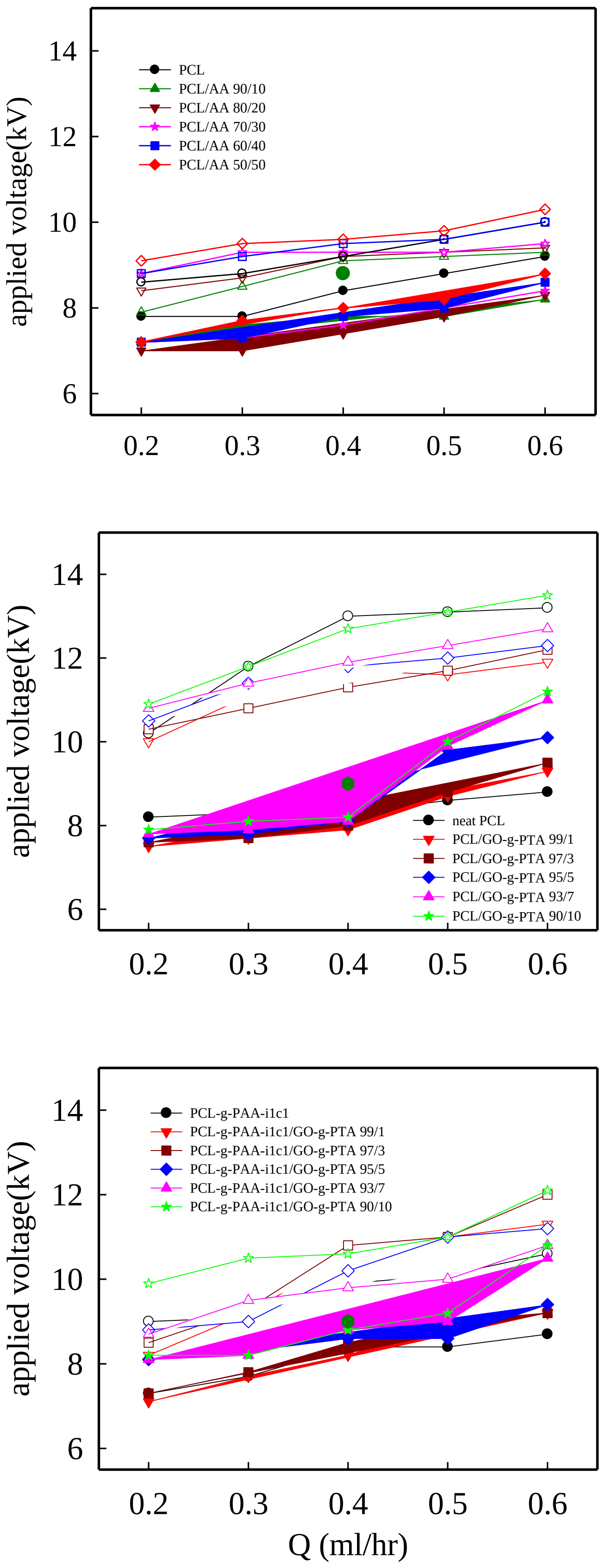

Figure S1. Functional domain for the electrospinning solution of 14 wt% PCL with (a) various AA contents, (b) various GO-*g*-PTA contents, and (c) 20 wt% AA and various GO-*g*-PTA contents. The domains indicated the range of operating electrical fields required for the stable cone-jet mode. (Filled symbols for lower bond applied voltage and open symbols for upper bond applied voltage).

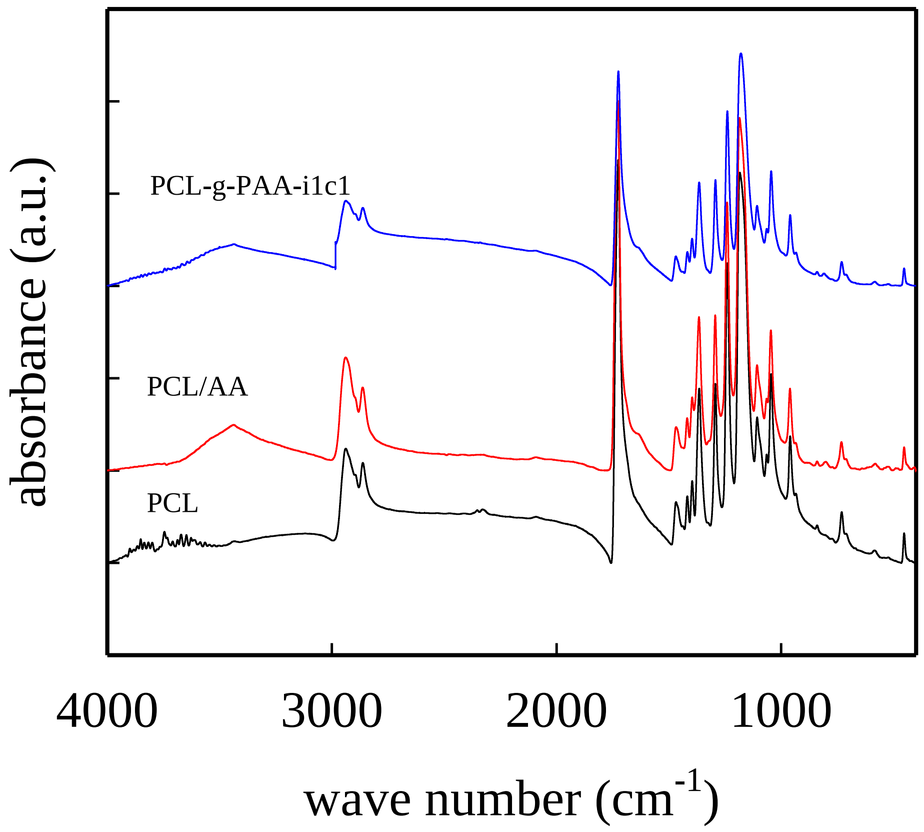

Figure S2. FTIR spectra of the electrospun PCL, PCL/AA 80/20, and PCL-*g*-PAA-i1c1 nanofibers.

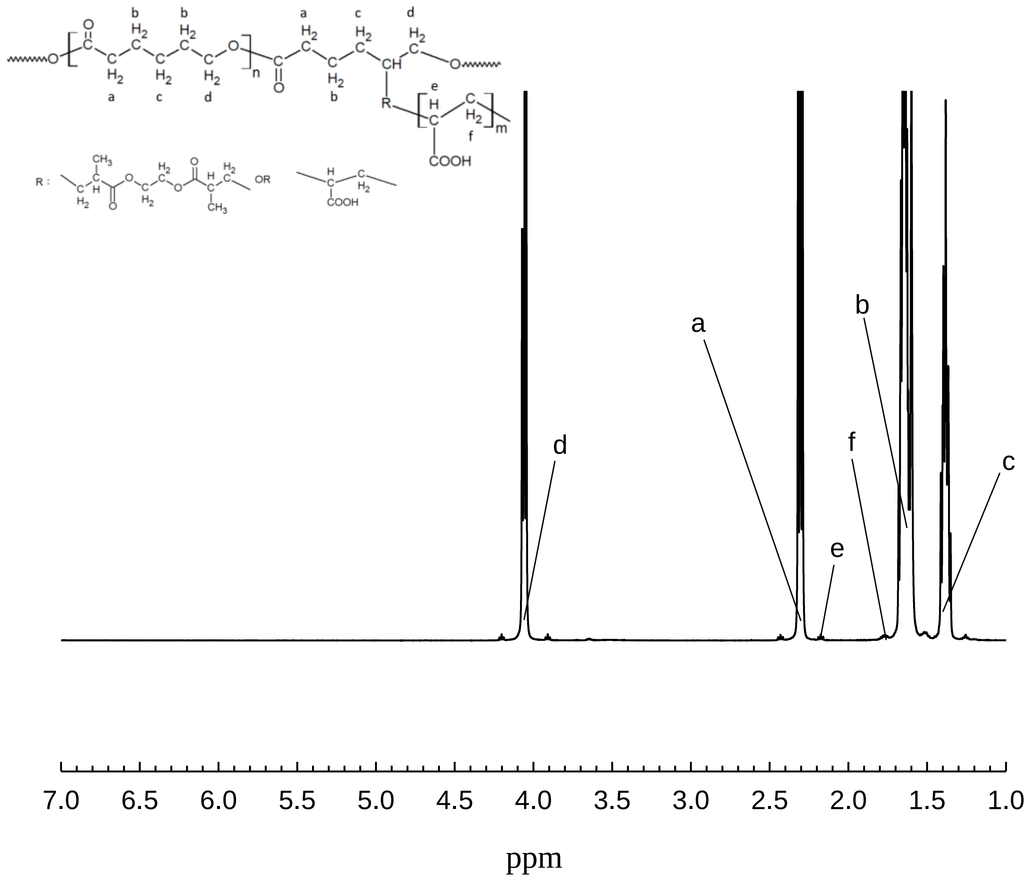

Figure S3. ^{1}H-NMR spectra of PCL-*g*-PAA.

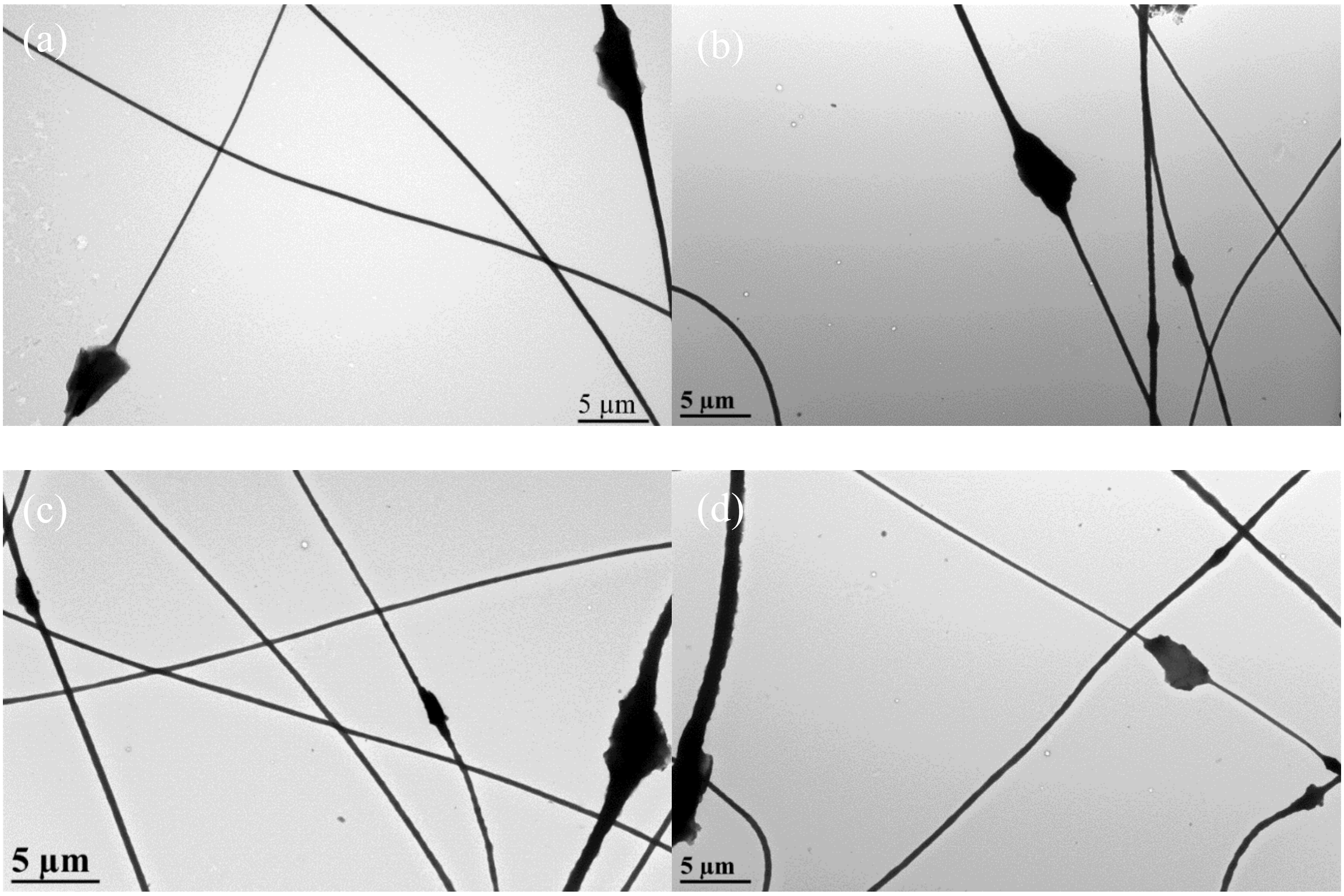

Figure S4. TEM images of electrospun PCL and PCL-g-PAA composite nanofibers filled with various amounts of GO-*g*-PTA. (a) PCL/GO-*g*-PTA 95/5, (b) PCL-*g*-PAA-i1c1/GO-*g*-PTA 95/5, (c) PCL/GO-*g*-PTA 90/10, and (d) presented the image of PCL-*g*-PAA-i1c1/GO-*g*-PTA 90/10.
